# Participants’ Evaluation and Outcomes following Integration of Self-Management Support into Outpatient Schizophrenia Case Management

**DOI:** 10.3390/ijerph20043035

**Published:** 2023-02-09

**Authors:** Heather E. McNeely, Lori Letts, Mary-Lou Martin, Susan Strong

**Affiliations:** 1 Schizophrenia and Community Integration Service, St. Joseph’s Healthcare Hamilton, Hamilton, ON L8N 4A6, Canada; 2 Department of Psychiatry & Behavioural Neurosciences, McMaster University, Hamilton, ON L8N 3K7, Canada; 3 School of Rehabilitation Science, McMaster University, Hamilton, ON L8S 1C7, Canada; 4 Forensic Service, St. Joseph’s Healthcare Hamilton, Hamilton, ON L8N 4A6, Canada; 5 School of Nursing, McMaster University, Hamilton, ON L8S 4L8, Canada

**Keywords:** mental health, schizophrenia, self-management, prevention and early intervention, psychosocial care, lived experience, self-care

## Abstract

(1) Background: Self-management is advocated as a feasible, effective intervention to support individuals to actively manage the impact of illness and live healthier lives. We sought to evaluate a piloted self-management model, SET for Health, tailored for individuals living with schizophrenia embedded within ambulatory case management. (2) Methods: A mixed-methods design engaged 40 adults living with schizophrenia in the SET for Health protocol. Functional and symptomatic outcomes were measured by self-report and clinician ratings at baseline and completion of self-management plans, on average one year later. Semi-structured qualitative client interviews invited evaluations of personal experiences with the intervention. (3) Results: Significant improvements were found concerning client illness severity, social and occupational functioning, illness management and functional recovery with reductions in emergency visits and days in hospital. Clients endorsed the value of the intervention. Baseline clinical characteristics did not predict who benefited. Participation contributed to motivational gains and quality of life. (4) Conclusions: Results confirmed self-management support embedded within traditional case management improved clients’ clinical and functional status, and contributed to quality of life. Clients engaged in their recovery and actively used self-management strategies. Self-management can be successfully adopted by clients with schizophrenia regardless of age, gender, education, illness severity or duration.

## 1. Introduction

It is widely accepted that embedding self-management support within traditional healthcare yields significant patient and system benefits for people with a variety of long-term health conditions [1,2]. Self-management support services assume a complex sequence of events and effects with multiple objectives and multiple endpoints for evaluation [3]. Services are expected to change participants’ health behaviours by increasing participants’ self-efficacy and self-knowledge, which is thought to lead to better illness control and improved health outcomes, leading in turn to well-being and the effective utilization of health services, particularly a reduction in emergency services and hospitalisations. In light of these benefits, health quality agencies have advocated for the inclusion of self-management supports within traditional medical models of care for individuals living with chronic mental health conditions, such as schizophrenia [4]. SET for Health, which stands for Self-management Engaging Together (SET) for Health, is a novel collaborative model of self-management support that was co-developed with client and provider input, and tailored to meet the needs of adults living with schizophrenia and receiving care within a traditional outpatient case management medical model of service [5,6]. The process of development and integrating this model of care within existing mental healthcare resources is described in detail in previous papers by our group [5,6]. In this paper, we describe the clinical, social, and functional outcomes following a one-year trial of SET for Health.

Schizophrenia is a complex, typically chronic mental health condition, that affects about 1% of the population, characterized by positive (hallucinations and delusions), negative (social withdrawal, flat affect) and cognitive (attention, memory, executive functions) symptoms with onset in the late teens to early adult years [7]. Individuals with schizophrenia experience a higher incidence of multiple co-occurring physical health conditions even after controlling for factors such as age, gender and social determinants of health [8]. For this group, the ability to actively self-manage health and well-being is a key component to successful recovery and prevention of relapse [4,9,10] where recovery refers to “living a satisfying, hopeful and contributing life, even when a person may be experiencing ongoing symptoms of a mental health problem or illness” [11]. However, in traditional healthcare settings, individuals living with schizophrenia are often insufficiently engaged in their own treatment process [12]. Self-management support is advocated as a feasible, effective intervention for building capacity with individuals/families to actively manage the impact of illness and live fuller, healthier lives. Self-management interventions offered to individuals with schizophrenia and serious mental illness are typically educational; these interventions are usually delivered alongside treatment as usual, and involve providing information about mental illness and how it is treated, identifying warning signs and developing relapse prevention plans, and practical skill-building around coping with residual symptoms of mental illness within a recovery-oriented framework [12,13]. Systematic reviews and meta-analyses indicate that this format of self-management support for individuals with schizophrenia and serious mental illness is associated with improved outcomes including reduced psychiatric symptom severity, and when self-management is broadened beyond illness management to focus on personal and functional recovery, interventions also offered functional benefits as well as improvements in self-reported recovery and self-efficacy [12,13]. Despite positive indicators based on results of meta-analyses, there is variability in specific methods of delivery and outcomes measured and questions remain about how best to deliver self-management support with this population.

Bodenheimer, McGregor and Sharifi [14] identified self-management support as a portfolio of techniques and tools focused on choosing healthy behaviours that also involves a transformational collaborative patient–caregiver partnership. In this light, self-management support is more than patient education or life skills training. The delivery of self-management support involves a fundamental culture transition in healthcare organizations [5,6]. To address issues of access and meaningful delivery, a group of clinician researchers, clients and service providers co-designed and implemented SET for Health, a tailored model of self-management support within a personal recovery framework deliberately embedded into routine case management schizophrenia services [5]. Core components of standardized self-management programs were synthesized into a model for feasible delivery on an individual basis within existing clinic resources. SET for Health was designed around five core essentials and involved motivational elements [5]. The pursuit of personal recovery goals in the context of managing life challenges was the framework. The delivery of SET for Health was embedded into regularly scheduled care. As such, sessions and delivery of the intervention varied depending on the individual patient’s care schedule and fluctuating acuity of care needs, ranging from twice per week to once every three weeks. During visits with their case manager, clients were coached to actively participate and reflect on experiential learning. Interprofessional case managers (registered nurses, social workers, occupational therapists) used elements of motivational interviewing, cognitive behavioural strategies, and principles of adult education in applying the intervention. Driven by each client’s recovery goals in the context of daily living challenges, SET for Health followed a targeted approach involving the creation of client–provider collaborative spaces to co-construct personal self-management reference tools, that coupled with organisational changes of care delivery, culminated in the co-development of a self-management plan. The completion and active implementation of the self-management plan by clients represented completion of the study protocol [5]. Further details about the intervention delivery, research design and methods are described separately [6] as is the background processes of d developing the concept and preparing the site at a tertiary, public mental health centre in Ontario, Canada [5].

In this paper, we examine whether the implementation of the SET for Health trial was successful in terms of client outcomes and perspective, and we explore who may benefit most from this intervention. The questions asked: Was participation in SET for Health associated with meaningful clinical and functional changes for clients living with schizophrenia? Did any individual differences or clinical features at baseline prevent or enhance participation in SET for Health, such as level of education, age, psychological factors (e.g., motivation, confidence) or illness chronicity? From the client’s perspective, based on experiences with the new model of delivering self-management support, to what extent did SET for Health offer added value to usual care?

## 2. Materials and Methods

Findings presented here are drawn from a mixed-methods study in which the quantitative component was nested within the primary qualitative component to understand and evaluate how the model of delivery worked in the practice context [6]. Nine inter-professional case managers trained in the provision of self-management support [6] offered SET for Health to a convenience sample of clients living with schizophrenia from their caseloads.

Prior to data collection, the study received Research Ethics Board approval (Hamilton Integrated Research Ethics Board #3733), and informed written consent from participants was obtained with rolling entry from September 2017 to July 2019. Participants received gift cards for their involvement in the study. Completion of the SET for Health protocol was set at the point when the last required tool, the self-management plan, was completed and being implemented in client’s lives [6]. The self-management plan represented a summary of personal learnings regarding wellness strategies, illness management and crisis planning. At baseline within one-week pre intervention and at post intervention within one month following completion of the protocol, client participants completed study measures, as well as post-intervention interviews with the principal investigator (SS), a registered occupational therapist clinician-researcher employed by the healthcare centre but not directly involved in client care. Pre and post intervention, clients’ case managers completed two measures of client participants’ functional and self-management status and clients’ psychiatrists rated clients’ illness severity. Specific measures completed by clients, case-managers and psychiatrists are described in Section 2.2.

### 2.1. Sample

The sample was drawn from two outpatient programs mandated to deliver mental health and addiction services to individuals with schizophrenia and related psychotic disorders and co-morbidities living in the community within a large urban and rural catchment region. Clients were eligible to be recruited into the study if they were English speaking, registered with either program and were receiving care from one of the providers participating in the initiative. Potential client participants were excluded if they were unable to give informed consent (i.e., designated treatment incapable by treatment team), or had such severe cognitive impairment they were unable to complete the study measures or intervention tools.

As described previously [6], we used a confidence interval approach [15] to set the sample size based on the proportion anticipated to complete self-management plans at 12 months. We used a benchmark of ≥44% of the intent-to-treat sample completing the self-management plan as an index of successful delivery of SET for Health, based on successful completion rates of the Illness Management and Recovery Scale in previous research with a similar clinical sample [16,17]. As reported in our prior feasibility paper [6], with our intent-to-treat sample of 51 consenting clients, we exceeded the ≥22 completed self-management plans required to validate delivery of the intervention. We also exceeded the ≥51% retention rate benchmark [6]. On average, most clients required a full year to complete the SET for Health protocol (M = 12.8 months, SD = 4.9 months) [6].

### 2.2. Quantitative Measures

#### 2.2.1. Client Self-Report Measures

The Illness Management and Recovery Scale (IMR)—Self version [18,19] includes 15 items measuring illness self-management and pursuit of recovery goals for treatment planning. It was developed for use with the IMR program in a population similar to our study target population. Recommended for tracking outcomes from participating in other self-management interventions, it has moderate internal consistency and high 2-week test re-test reliability (r = 0.81); it has also been correlated with self-ratings of recovery and symptoms.

The Sense of Coherence (SOC) [20] is a 29-item global measure of sense of coherence (SOC), i.e., internal resilience that promotes health in adversity involving three subscales: (a) ‘comprehensibility’ or ability to perceive situations as clear, structured; (b) ‘manageability’ or belief of holding necessary skills to deal with life challenges; and (c) ‘meaningfulness’ or confidence that life challenges are worthy of investment and engagement. Designed to predict and explain movement towards health according to the salutogenic model, a health promotion framework that focuses on peoples’ internal resources to deal with environmental perceptions and life challenges, the SOC is used internationally in both healthy and ill populations.

The Patient Activation Measure for Mental Health (PAM-MH) [21] is a 13-item scale that measures a person’s knowledge, skills and confidence to manage illness, in particular mental health. Scores range from 0 (least activated) to 100 (most activated). The scale has good test–retest reliability [22] and has been used with schizophrenia samples (e.g., [23]).

The Integrative Hope Scale (IHS) [24] is a 23-item 6-point Likert scale (strongly disagree to strongly agree) with four dimensions: ‘trust and confidence’, ‘positive future orientation’, ‘social relations and personal value’ and ‘lack of perspective’. It has been used with individuals living with schizophrenia with good test–retest reliability and internal consistency [24].

The Self-Rated Health Scale (SHS) provides an overall health rating on a single item scale using a semantic differential from 1 (excellent) to 5 (poor) in response to question: “In general, how would you say your health is?”. This scale has been used with older adults with schizophrenia [25] and chronic illness populations [26], with good prediction of mortality [27].

The Quality of Life Enjoyment & Satisfaction Questionnaire (QLES-Q) [28] is a 22-item scale using a 5-point semantic differential from 1 (not at all or never) to 5 (all the time or frequently). It was developed as a general quality of life measure with both psychiatric and general populations, with high internal consistency and stability and predictive validity of domains (physical health, feelings, leisure activities, social relationships) demonstrated in samples of individuals with schizophrenia and mood disorders [29].

#### 2.2.2. Case Manager Evaluations of Client Participants

The Social and Occupational Functioning Assessment Scale (SOFAS) [30] is a standard measure with high inter-rater reliability in a clinical practice with schizophrenia. The overall severity of psychiatric disturbance with respect to social and occupational functioning across daily life activities is rated with a single score (1–100) on a continuum from excellent functioning to grossly impaired functioning, including physical and mental impairments where higher scores indicate better functioning.

The Illness Management and Recovery Scale (IMR)—Clinician version [19] includes 15 items measuring illness self-management and the pursuit of recovery goals for treatment planning. The clinician version mirrors the self-report, client version. The convergent validity of the clinician version correlated with clinician ratings of community functioning.

#### 2.2.3. Psychiatrist Evaluations of Client Participants

A Clinical Global Impression Scale—Illness Severity [31], is used as a global impression, rating severity of psychopathology over the past week based on all information available on 7-point semantic differential scale from 1 = normal, not at all ill to 7 = among the most extremely ill patients. Global impression ratings have been shown to correlate well with other standardized measures of psychiatric symptom severity [31].

### 2.3. Qualitative Evaluation Methods

For the purposes of this study, learning about self-management by participating in SET for Health was defined as the process of learning about your own health and what you can do to manage your condition and live well. Semi-structured interviews with clients lasting 40 to 60 min were completed post intervention by the study’s principal investigator (SS), a registered occupational therapist and clinician-researcher employed by the hospital program but not directly involved in client care. An interview guide was used to provide a consistent framework of questions; the exact order and language used varied by participants in order for the interview to be participant-directed and to support the participant telling their own story of their experiences with SET for Health, what they took away from that experience, any impact of the experience on themselves, and recommendations for future delivery. Interviews were audio-recorded with participant consent. Participants’ summative comments, evaluations and recommendations for delivery from the post interviews are briefly described in this paper. More detailed analyses of participants’ experiences with SET for Health, with a focus on the meaning of the experiences, as well as the full interview guide, will be shared in future phenomenological papers.

### 2.4. Data Analysis Methods

#### 2.4.1. Quantitative

Statistical analyses were conducted using IBM SPSS Statistics 28.0.1.0 software (Copyright IBM Corporation, licenses 1989, 2001; www.ibm.com (accessed on 1 February 2023); IBM Canadian Office, Markham, Ontario). Descriptive statistics were completed to describe the client sample. A series of one-tailed paired samples *t*-tests were conducted to assess a priori hypotheses regarding anticipated improvements in clinical, functional and quality of life measures from pre to post SET for Health. Two-tailed Pearson correlations were calculated to explore relationships among variables pre and post intervention. A series of linear regression analyses were completed to determine whether baseline psychological or clinical characteristics or individual differences could predict which participants were best able to participate in SET for Health, and whether degree of participation in SET for Health predicted benefits as measured by changes in clinical, functional or quality of life measures from pre to post intervention. Outcome measures included: participation in SET for Health as measured by change in client IMR-Self score; Clinical Mental Health Status, as measured by change in CGI-Illness Severity score; Functional outcome as measured by change in SOFAS score, and Quality of Life outcome, as measured by change in QLES-Q score.

#### 2.4.2. Qualitative

Emergent content analysis was selected as the best approach when analyzing complex, sensitive phenomena, and when interested in manifest content while intending for a low level or surface interpretation [32] (e.g., relaying participants’ voices in context at face value). Interview transcripts were read as a whole, with a particular focus on responses to questions asking for participants’ opinions about the model of delivery and recommendations. Using an editing analysis style [33] and iterative reflexivity, text was systematically grouped by pattern while writing general descriptions of each pattern, which were combined into larger trends and characteristics of context. Inconsistencies or critical comments were explored collaboratively by the co-authors who are all clinician-researchers not directly involved in care of study patients in order to fully understand the diversity and plurality of perspectives. The findings reflect a descriptive synthesis of content, grounded and illustrated with cogent quotations.

## 3. Results

### 3.1. Participants

A total of 51 eligible clients were recruited. Of the intent-to-treat sample, eleven clients did not receive the intervention: five were withdrawn for acute medical issues or significant substance use, one died of long-standing medical conditions, and five left the clinic. Therefore, 78.4% (40/51) of participants (18 male, 22 female) received the intervention with 21.6% (11/51) attrition from all causes. Three participants did not complete the protocol within the one-year study period due to medical issues interrupting delivery for a 72.5% (37/51) completion of self-management plans.

Clients who received the intervention (*n* = 40) represented a chronically ill, ambulatory client population (see Table 1). On average, they were middle-aged (mean = 46.6 years, SD = 12.61) with a high school education (mean =13.3 years, SD = 2.8 years) and had lived with their illness for an average of more than 20 years (years diagnosed: mean = 21.9 years, SD = 12.5 years). They comprised 18 self-identified women and 22 men ranging in age from 22 to 72 years. Co-occurring medical and substance use issues, as well as social barriers to health were common among the sample. Of the study completers, 68% were being treated for physical co-morbidities and 30% were noted to have substance use issues (alcohol, cannabis, poly-substance). Reflecting the illness severity, social and occupational challenges in a diverse sample, 28% were on Community Treatment Orders (a provision under the Ontario Mental Health Act that allows for a physician to mandate treatment in the community), 25% had a history of involvement with courts and 20% reported a history of homelessness. At the time of the study, 40% resided in assisted living facilities, 30% cohabitated with family/partner, and 28% were living alone in the community. A third (32%) of the sample reported engaging in regular care-giving responsibilities for family members (e.g., children, aging parents, grandparents). The majority (80%) of the sample was unemployed or retired and were supported by a combination of disability and social benefits and family savings on entry to the study. However, 10% of clients reported regular part-time employment and another 10% reported casual or occasional work.

### 3.2. Observed Functional and Clinical Outcomes

Functional improvements following completion of SET for Health was noted in returning to work and meaningful activities. Two client participants who had been on long-term sick leave from employment returned to full-time work following completion of SET for Health. Three of four student participants who had placed courses on hold returned to school and another four participants enrolled or began formal academic courses (grade 12 equivalency, college and university). One client had been actively volunteering on entry to the study, and this number increased to five client participants participating in formal volunteer activities in the community after completion of SET for Health. These observations are supported by a statistically significant increase in self-reported time spent in structured roles (i.e., working, volunteering, studying, care-giving), as measured by the IMR Scale item 5 from pre to post SET for Health, *t*(34) = −2.9, *p* = 0.003.

Further evidence supporting functional and clinical improvements following the intervention is highlighted in the striking reduction in mental health emergency department visits, hospitalisation rates and duration of hospitalisations when comparing usage in the one-year pre versus one-year post participation in SET for Health (see Table 2).

### 3.3. Measures of Clinical and Social Functioning

Scores on measures completed by clients, case managers and psychiatrists at baseline and post SET for Health are included in Table 3.

#### 3.3.1. Correlations among Measures Pre–Post SET for Health

At baseline, prior to SET for Health, there were significant negative correlations between lower scores on clients’ self-rated health (SHS, reflecting better self-reported health) and higher client-rated scores on resilience (SOC; r = −0.589, *p* = 0.01), activation (PAM-MH; r = −0.613, *p* = 0.01), and hope (IHSS; r = −0.570, *p* = 0.01). Similarly, there were significant positive correlations between client-rated quality of life (QLES-Q) and clients’ ratings on SOC (r = 0.591, *p* = 0.01), PAM-MH (r = 0.594, *p* = 0.01) and IHSS (r = 0.811, *p* = 0.01). Higher baseline levels of hope (IHSS) were positively correlated with higher level of resilience (SOC; r = 0.736, *p* = 0.01) and activation (PAM; r = 0.673, *p* = −0.01), and SOC and PAM were also correlated with each other (r = 0.616, *p* = 0.01). Therefore, going forward, we chose to refer to these three variables (SOC, PAM-MH and IHSS) as ‘motivational factors’.

At baseline, there were no significant correlations between provider-rated baseline function (SOFAS) scores or psychiatrist-rated baseline illness severity (CGI-IS) scores and any of the client-rated baseline health, quality of life or motivational factors.

Following participation in SET for Health, better client Self-Rated Health Scale scores continued to be significantly correlated with higher ratings on the ‘motivational factors’ SOC (r = −0.546, *p* = 0.01), PAM-MH (r = −0.440, *p* = 0.05) and IHSS (r = −0.494, *p* = 0.01) but were also newly and positively correlated with psychiatrists’ ratings of overall illness severity (r = 0.381, *p* = 0.05), indicating that both clients and psychiatrists had shared opinions of their overall health/mental health status following SET for Health. Post-SET for Health, clients’ ratings of quality of life (QLES-Q) continued to be positively correlated with higher ratings of SOC (r = 0.721, *p* = 0.01), PAM-MH (r = 0.561, *p* = 0.05) and IHSS (r = 0.860, *p* = 0.01) but were newly correlated with better scores on Self-Rated Health Scale (r = −0.539, *p* = 0.01) and with provider ratings on the SOFAS (r = 0.445, *p* < 0.001). These findings suggest that after SET for Health, clients perceived a greater relationship between both their health and their quality of life, and that their quality of life was now correlated with measures of social and occupational functioning (SOFAS).

Following SET for Health, client ratings of hope (IHSS) continued to be positively correlated with client higher ratings on resilience SOC (r = 0.821, *p* = 0.01) and activation PAM (r = 0.672, *p* < 0.001), but were now also positively correlated with providers’ function (SOFAS) ratings (r = 0.485, *p* = 0.01). SOC and PAM continued to be correlated with each other (r = 0.697, *p* = 0.01).

#### 3.3.2. Pre–Post IMR Self and Clinician Outcomes: Participation in SET for Health

Statistically significant improvements were found in both client and provider ratings on the Illness Management and Recovery (IMR) Scale (client ratings, *t*(34) = −7.59, *p* < 0.001; provider ratings, *t*(29) = −8.34, *p* < 0.001). Client and provider IMR ratings did not differ significantly from each other either at baseline or following completion of the self-management plan (see Table 3).

#### 3.3.3. Pre–Post Clinical, Functional and Quality of Life Outcomes

Significant improvements following SET for Health were found on the psychiatrist-rated CGI Illness Severity scale, *t*(32) = 3.14, *p* = 0.002 and on the case manager-rated SOFAS total Social and Occupational Functioning score, *t*(29) = −5.1, *p* < 0.001. While mean scores on the client-rated QLES-Q increased slightly from baseline to follow-up, this difference was not statistically significant.

#### 3.3.4. Predicting Pre–Post SET for Health Outcomes

A series of three linear regression analyses were conducted to predict who would benefit most from SET for Health using baseline factors grouped conceptually as follows: (1) Motivational Factors, as measured by baseline PAM, Hope Scale and SOC (activation, hope, resilience); (2) Clinical Factors, as measured by baseline CGI-IS, SHS, SOFAS (illness severity, self-rated health, social and occupational functioning); (3) Individual Difference Factors, as measured by age, years of education, tenure with provider, and years since onset of psychotic illness. Outcome measures included: Participation in SET for Health as measured by change in client IMR score; Clinical Mental Health Status, as measured by change in CGI-IS score; Functional outcome as measured by change in SOFAS score, and Quality of Life outcome, as measured by change in QLES-Q score.

Baseline motivational factors (PAM, SOC, IHSS scores) together significantly predicted level of participation in SET for Health as measured by change in client IMR ratings, *F*(3,27) = 4.55, *p* = 0.011, accounting for 26% of the variance in IMR change score (Adjusted R^2^ = 0.26). Baseline clinical or individual difference factors did not reliably predict IMR outcomes.

None of the regression models using Motivational, Clinical, or Individual Difference factors reliably predicted change in Clinical, Functional or Quality of Life outcomes following completion of SET for Health protocol.

**Figure 1:**
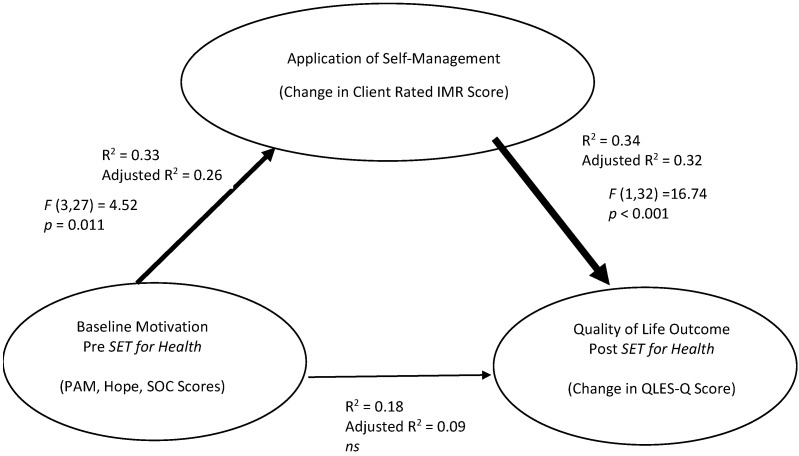
Participation in SET for Health as measured by improvement in client-rated IMR score mediates the relationship between baseline client motivation factors and improvements in client-rated quality of life outcome.

However, the degree of participation in SET for Health predicted a significant proportion of the variance in Quality of Life Outcomes. A change in client IMR score accounted for 32% of the change in QLES-Q score, *F*(1,32) = 16.74, *p* < 0.001 (Adjusted R^2^ = 0.32). These findings suggest that participation in self-management (change in IMR-Self score) mediated the relationship between baseline motivational factors and quality of life outcomes (see Figure 1).

### 3.4. Qualitative Client Evaluation

In keeping with statistically significant improvements on the client-rated IMR scale, in general, clients described SET for Health as having supported their engagement and participation in care and recovery, promoted learning about self-management and themselves, facilitated open communication, and fostered increased self-confidence to manage life challenges. When client participants were asked: ‘So what did you think about all this SET for Health?’ with the client’s reference tools spread in front of them, the products of the SET for Health intervention, their summative comments reflected the range and various qualities of participation:

“I think it was a good… Like, it got me more motivated to do my goals and stuff because I had organization and I had a direction… I followed my goals and my routine and everything. I’ve made progress… I learned what stresses me out. Not doing my goals. And that’s why we configured the plan around that… Yeah, I was going to say that this whole experience, filling out these questionnaires and reports basically just helped me figure out what triggers me and how to deal with problems coming my way… I think it’s about finding yourself and what’s important to you.”

(C016)

“It was helpful… I just know it was helpful to write it all down. Yeah just to make it plain… Makes it plain and honest… honest and real. Yeah… That does help with the communicating.”

(C048)

“I mean I felt very helpless before and now I feel you know I might have bad days or weeks or months or… the Friend’s theme song comes to mind… There’s a lot of understanding being developed. There’s a lot of techniques and medications and you know with so many tools. Like I’m not going to get despondent until I run out of tools and haven’t had any positive effect, right… And I’m already having lots of positive effects.”

(C041)

“… it gives them, the doctors and everybody more input into your care, right… like insight into what your care needs to be and all the different… ways that people need help. So, I think it’s a good thing yeah.”

(C042)

The voices above expressed qualities of their experiences participating in SET for Health as: motivating, activating, self-determining, self-discovering, communicating, hopeful, meaningful and insightful.

A few clients complained of “all these papers” and the work involved. Yet, when the interviewer empathized with them and then commented, “Maybe we shouldn’t be doing this then, putting clients through all this”, clients were very quick to correct the interviewer. When the interviewer pressed for an evaluation, they responded:

“It’s a good life inventory”.

(C034)

“I’d say it’s good for your health and there’s a positivity there.”

(C011)

When clients were asked: “To who do you think we should be offering SET for Health? Who do you think would benefit from the experience?”, client participants recommended inviting all clients to SET for Health, while expressing some doubts about all clients’ willingness or ability to engage.

“I think it would be helpful for everyone… I think everyone would learn a lot about themselves.”

(C017)

“Anybody… Yes, definitely. Everybody not just anybody everybody. I think people with… who don’t even have mental illnesses could benefit from something like this… I’m sure I’m not the only success story but if it could help you could definitely use my life as an example. I’m sure I’m not the only success story.”

(C014)

“I don’t think it’s for everybody. I don’t think everyone has the patience or the mentality to do it but those who do should probably go through it. Or at least give them the chance before you say that you can’t do it, because they could just turn around, and say at one point no, I don’t feel like doing this anymore. Then at least you gave them the chance. But if you get through it, you can learn a lot.”

(C047)

## 4. Discussion

A sample of adults receiving services for schizophrenia/schizoaffective disorder and co-morbidities successfully completed SET for Health, a targeted, collaborative self-management support program embedded within a standard outpatient case management service. SET for Health was designed around five core essentials and involved motivational elements [5]. The pursuit of personal recovery goals in the context of managing life challenges was the framework. Clients were coached to participate and reflect on experiential learning opportunities. Providers used elements of motivational interviewing, cognitive behavioural strategies, and principles of adult education. Further reference tools were co-created and tailored in client–provider partnerships. This paper built upon an earlier report of how this novel model demonstrated feasibility with retention and completion rates exceeding predetermined criteria based on the literature [6]. A preliminary evaluation of client interviews conducted post intervention found that clients reported participation in SET for Health to be valuable and that they saw benefits to their participation in their own care and in their pursuit and achievement of daily living goals, and this was reflected in statistically significant improvements on both client and provider IMR scores. Moreover, completion of the intervention was also associated with statistically significant improvements in provider-rated clinical and social functioning and with significant increases in clients reporting time spent in structured community-based activities. Some clients returned to work/school and others increased participation in school, formal community volunteer activities and other caregiving roles. The impact of these clinical and functional improvements is underscored by striking reductions in hospital admissions and emergency room visits for mental health concerns from one year before to one year following participation in SET for Health. Together, these findings indicate that a formal self-management support intervention purposefully embedded in case management services, such as SET for Health, may be an effective element of treatment and may support meaningful recovery with this at-risk mental health population.

Importantly, there were no statistically significant differences in client versus care provider IMR ratings, reflecting a shared understanding of clients’ perceptions and implementation of self-management behaviours. This may be one indication that delivery in the SET for Health model involved the creation of collaborative client–clinician spaces for self-reflection and meaningful dialogue. Such collaborative learning spaces contrast with criticisms of other self-management interventions for not being delivered within a recovery-oriented approach or for restricting focus to a set of prescribed learning modules rather than addressing clients’ life challenges [34,35]. Increased client–provider collaboration following SET for Health may also be reflected in the better alignment between client and provider ratings of health, illness severity and social and occupational functioning that were not present at baseline. There is substantial research establishing that client–provider shared decision-making and personalized care planning delivered in a collaborative process of goal-setting and action-planning leads to self-management capabilities and behaviours, and better physical and mental health outcomes. These findings of improvements with increased collaboration are particularly striking when the intervention is comprehensive, intensive and integrated into routine care [36]. These were important considerations underlying the development of SET for Health, which was purposefully integrated into routine care with clients’ own case manager delivering self-management support as an augmentation to pre-existing service delivery [5].

Another important finding to emerge from this study relates to decision-making regarding which clients should be offered self-management support. Prior to, and even following some successes in delivery of self-management support, some provider participants held beliefs that certain clients (e.g., those with more severe illness, substance use, lower levels of education or lengthy tenure under more medicalized traditional case management) might be less interested in participating in SET for Health, and may not be successful [6]. Contrary to these beliefs, both our preliminary qualitative reports from clients as well as our quantitative findings suggest that self-management support should be offered to any and all clients regardless of age, education level, illness severity or chronicity, or length of engagement in traditional medical models of care; none of these factors significantly predicted clients’ abilities to engage and participate in the SET for Health intervention.

The quantitative results also reveal some intriguing relationships among client variables that do mediate certain outcomes. Despite significant improvements post intervention on illness severity and social and occupational function ratings by psychiatrists and case managers for the sample as a whole, clients’ self-rated health, activation, level of distress, hope, sense of coherence and ratings of their quality of life generally did not improve significantly following SET for Health. This may suggest some degree of lack of insight or self-reflection related to psychological or motivational constructs as opposed to more outwardly observable changes, as clients did endorse significant improvements in their use of self-management actions and behaviours as reflected in significant changes in both client and care provider IMR ratings. It is also possible that other elements of clients’ circumstances that were not measured (e.g., social determinants of health) may significantly impact psychological and motivational characteristics and quality of life, or that improvements in quality of life require more time than allocated for this study. That said, results of mediation analyses suggest an interaction between baseline client motivational characteristics, participation in SET for Health and client-rated quality of life outcomes. Clients with higher levels of baseline motivational factors, including optimism, trust, confidence, resilience, activation and hope were more likely to make significant investment in SET for Health, as reflected by greater changes in IMR scores, which was then associated with greater improvements in client ratings of quality of life. In interviews, client participants voiced experiencing feelings of motivation, hope and self-determination. These findings are consistent with results of previous studies that found greater hope and recovery orientations were associated with greater involvement in self-management (e.g., [37]). Similar results with a schizophrenia population were reported by Zhou and Li [38] who found that motivation was significantly correlated with engagement in self-management. These authors found that neurocognition, self-efficacy, and motivation were all directly related to self-management, as well as having indirect relationships with the self-management outcome. In their study, better performance on measures of neurocognition was associated with higher levels of self-efficacy and motivation, which then positively impacted self-management. Self-efficacy also had both a direct impact on self-management, as well as an indirect effect via the influence of higher levels of self-efficacy on higher levels of motivation. These results point to a complex relationship between a number of baseline factors and self-management outcomes.

In the present study, client-rated measures of hope, activation and sense of coherence were all motivational factors in the model that predicted level of participation in self-management. Sense of coherence as a construct involves self-efficacy as it is conceptualized as an internal resilience resource that promotes health in adversity. Multiple international studies link sense of coherence to health, health related behaviour, well-being, depression, anxiety, and substance use. Sense of coherence is thought to be a characteristic that mediates outcomes [39]. For example, a person with a strong sense of coherence activates internal resources and adopts attitudes and behaviours which are functional for coping. Factor modeling with a student sample found sense of coherence and resilience, and optimism were highly related, but sense of coherence had a greater predictive value of psychological symptoms of distress [40]. Other work has identified significant correlations between hope and patient activation in a schizophrenia sample, and found that these characteristics are not related to client demographic factors [23]. Together, the present findings and the evidence from prior studies highlight the importance of offering self-management support to all clients, but also speak to the importance of addressing motivational characteristics within the delivery of self-management support as a key factor to improve the level of engagement which may have a particular impact on quality-of-life outcomes.

There are limitations to this study that are important to acknowledge. The delivery of SET for Health was purposefully embedded within existing care, with interprofessional case manager care providers, which is seen as a strength of the intervention; however, this may have also presented a selection bias. Case managers delivering the intervention were encouraged by the investigators to offer SET for Health to any and all patients on their case load. However, as described in our previous paper documenting the pros and cons associated with embedding SET for Health within existing healthcare resources [6], study investigators could not completely control for possible biases in case manager perceptions as to which patients may or may not benefit from the intervention or be able to participate in research. Similarly, given the differences in baseline professional experience across different care providers, it is possible that some case managers may have been less apt to focus on motivational or psychological approaches and focused more on behavioural or action-centred approaches in their delivery of the intervention. This will be evaluated further in a subsequent phenomenological paper focused on interviews completed with the study care providers. The study is also limited by lack of a control group. Patient participants received treatment as usual prior to and concurrent with the study intervention, thus, the sample represented a within group pre–post design. Replication with a randomized controlled trial comparing SET for Health against a treatment-as-usual control group is a recommended next step.

## 5. Conclusions

The results of this study indicate that SET for Health offered an accessible mechanism for client participation, engagement and voice in the management of their mental and physical health. The statistical findings disproved many preconceived healthcare provider notions about who would (or would not) be able to engage in self-management [6], provide pause to reflect on views and prompt providers to protect and foster collaborative spaces with all of their clientele. The finding that motivational characteristics such as optimism, trust, confidence, resilience, activation and hope mediated the relationship between level of participation in SET for Health and client quality of life outcomes highlights the importance of creating opportunities to clinically target these factors as a means to improve outcomes for clients with schizophrenia participating in integrated mental health care.

The present findings add to the case for the routine integration of self-management support into ambulatory care services for adults living with schizophrenia and other mental health disorders, consistent with the standards of care outlined internationally in treatment guidelines [4,11,41]. Clients’ comments illustrate that they valued the opportunity for self-reflection, learning, and the positive spaces created to work collaboratively with the healthcare team. Given the significant gains made within a client sample living with severe and persistent mental illness, the findings of this study indicate that self-management commands attention as an intervention option for high-risk clients with limited insight, negative symptoms, medical co-morbidities and impoverished quality of life. Further, a targeted model of individually delivered self-management support within a traditional case management service, such as SET for Health, offers an accessible and feasible option that warrants replication with further, larger scale research trials.

## Figures and Tables

**Table 1:** Clinical and demographic characteristics of the sample at entry.

	Retained (N = 40)	Dropped Out (N = 11)
Participant Age (in years)		
Mean (S.D)	46.6 (12.6)	41.5 (17.9)
Median	51.5	39
Gender		
Male	22	8
Female	18	3
Education (in years)		
Mean (S.D)	13.3 (2.8)	11.9 (1.6)
Median	13.5	12.0
Age of Psychosis Onset (in years)		
Mean (S.D)	18.8 (6.6)	16.4 (9.1)
Median	17	16.0
Time Since Diagnosis (in years)		
Mean (S.D)	21.9 (12.5)	19.0 (17.6)
Median	19	12
Community Tenure (in months)		
Mean (S.D)	32.4 (59.1)	7.6 (11.6)
Median	4	1
Tenure with Treatment Program (in months)		
Mean (S.D)	36.3 (49.8)	20.3 (32.4)
Median	11.5	2.8
Tenure with Provider (in months)		
Mean (S.D)	14.7 (33.0)	5.6 (7.2)
Median	4	2.5

**Table 2:** Hospital Utilization and Emergency Department Visits Pre and Post SET for Health (*n* = 40).

	1 Year Prior	1 Year Post
Mental Health		
Number of Emergency Department Visits	31	3
Number of Hospitalizations	36	1
Duration of Hospitalizations (in days)		
Total Days of Hospitalization for Sample	2446	40
Mean (S.D)	61.2 (86.8)	N/A
Median	9	N/A
Physical Health/General Medical		
Number of Emergency Department Visits	23	30
Number of Hospitalizations	6	7
Duration of Hospitalizations (in days)		
Total Days of Hospitalization for Sample	53	35
Mean (S.D)	1.32 (6.9)	1.0 (4.2)
Median	0	0

**Table 3:** Pre–Post SET for Health Changes in Clinical and Functional Measures.

Measure	Time 1: Pre M (SD)	Time 2: Post M (SD)	*t*(df)	*p*
Self-Management -Client IMR	50.35 (7.34)	58.60 (6.64)	*t*(34) = −7.59	*p* ≤ 0.001
Self-Management -Provider IMR	49.22 (7.08)	59.12 (6.56)	*t*(29) = −8.34	*p* ≤ 0.001
SOC Total	137.2 (30.16)	140.93 (25.98)	*t*(29) = −0.97	Ns
SOC Comprehensibility	47.67 (13.47)	49.10 (11.14)	*t*(29) = −0.75	Ns
SOC Manageability	49.63 (10.66)	50.37 (10.82)	*t*(29) = −0.49	Ns
SOC Meaningfulness	39.90 (10.69)	41.47 (8.17)	*t*(29) = −1.08	
PAM	40.56 (6.82)	40.85 (6.22)	*t*(33) = −0.26	Ns
Integrated Hope Scale (IHSS) Total	106.97 (16.10)	107.76 (17.22)	*t*(33) = −0.33	Ns
Self-Rated Health	2.47 (0.98)	2.36 (0.90)	*t*(31) = 0.62	Ns
Client Distress (IMR Q6)	3.20 (1.47)	3.43 (1.29)	*t*(34) = −1.28	Ns
Social and Occupational Functioning -Provider SOFAS	M = 51.64 SD = 9.95	M = 61.89 SD = 11.40	*t*(33) = −5.09	*p* = 0.000
Quality of Life Client QLES-Q	81.59 (14.50)	84.47 (17.68)	*t*(33) = −1.08	Ns
Illness Severity -Psychiatrist CGI	M = 3.73 SD = 0.93	M = 3.31 SD = 1.1	*t*(31) = 2.95	*p* = 0.006

Results of one-sided *t*-tests provided given a priori hypotheses of improvement following intervention; clients with missing data excluded from individual analyses. IMR = Illness Management Recovery scale; SOC = Sense of Coherence; PAM = Patient Activation Measure; SOFAS = Social and Occupational Functioning Assessment Scale; QLES-Q = Quality of Life Enjoyment & Satisfaction Questionnaire; CGI = Clinical Global Impression.

## Data Availability

The data presented in this study are available on request from the corresponding author. The data are not publicly available due to healthcare privacy concerns.
